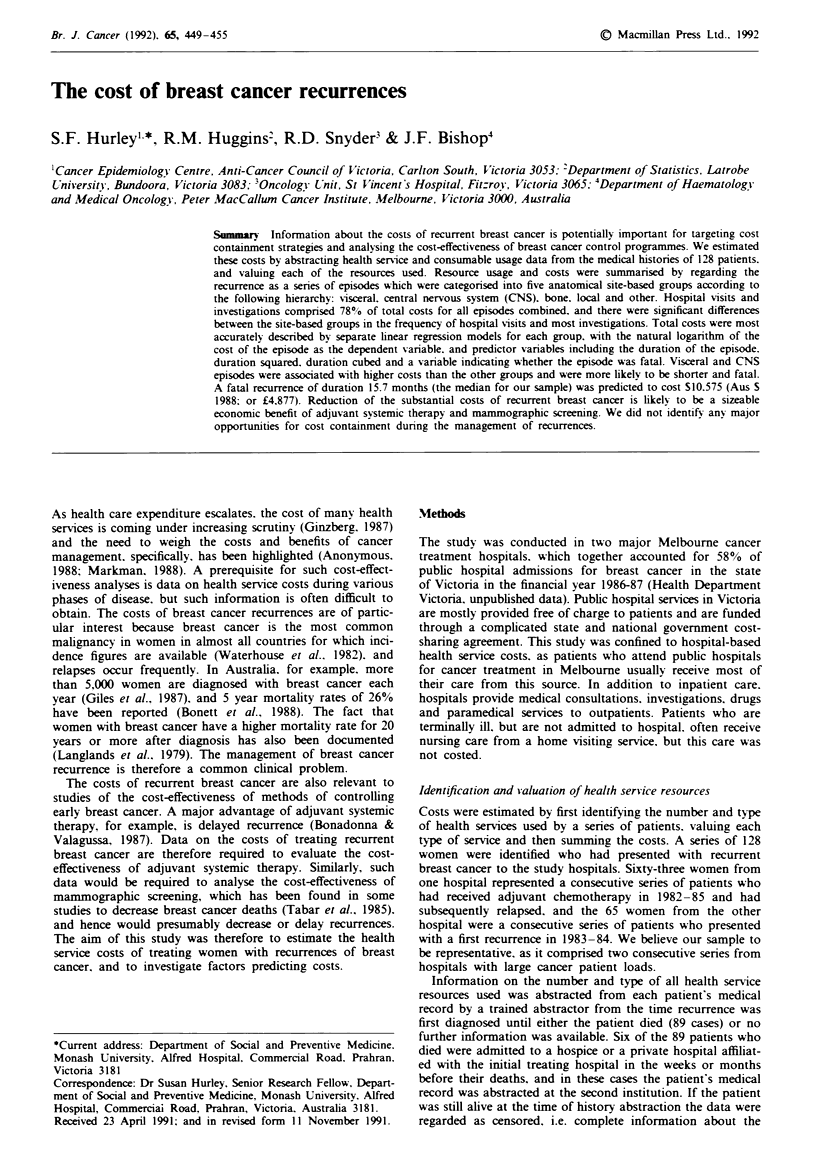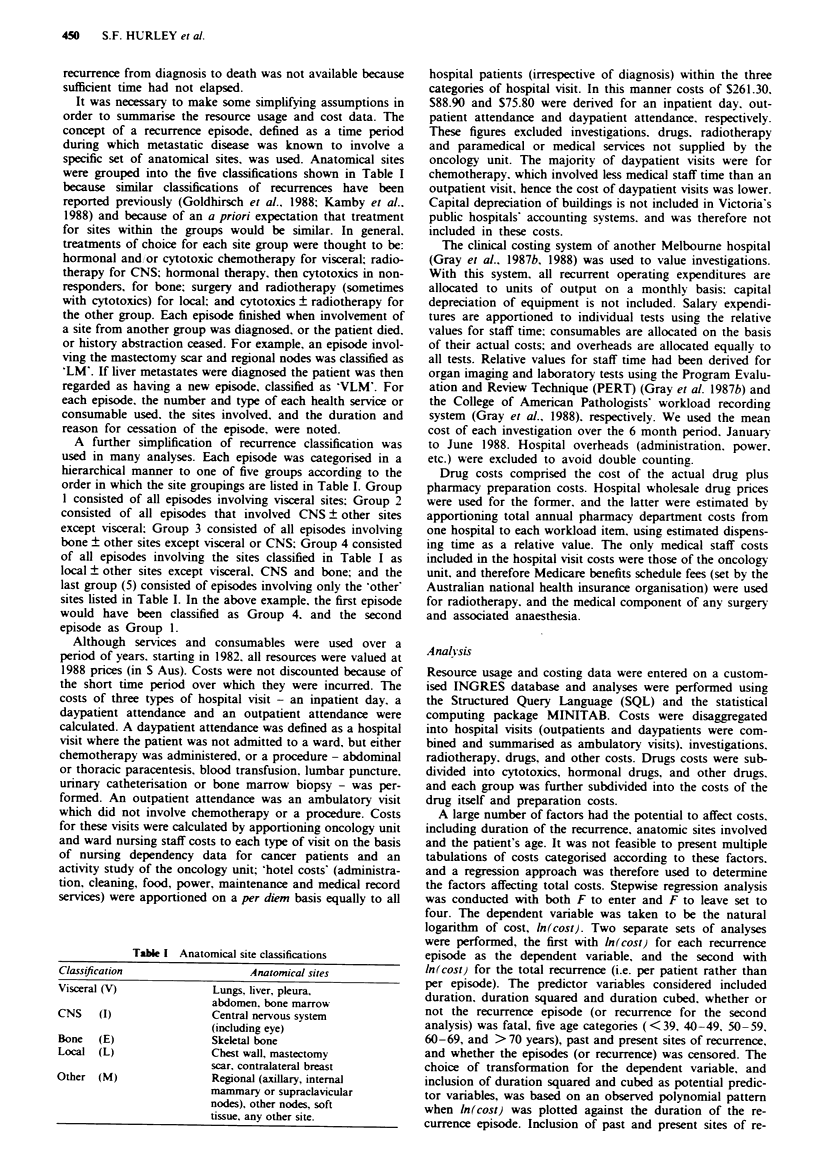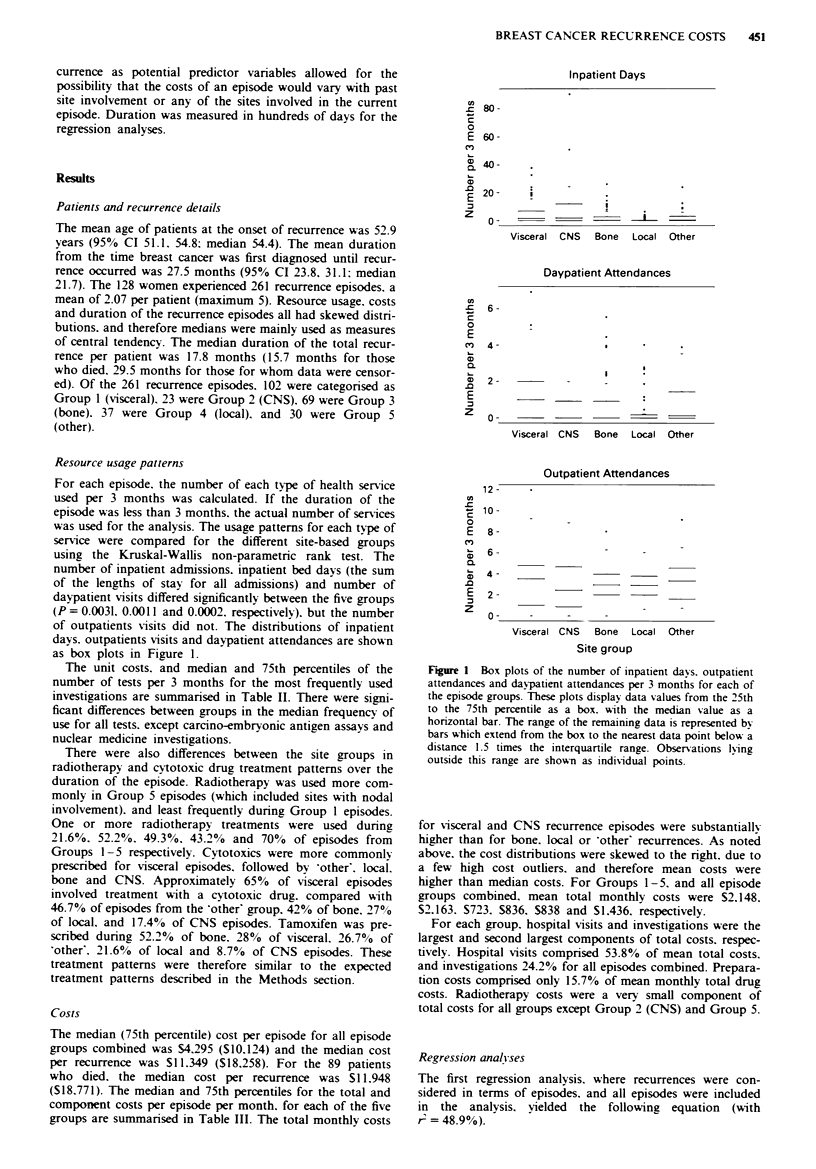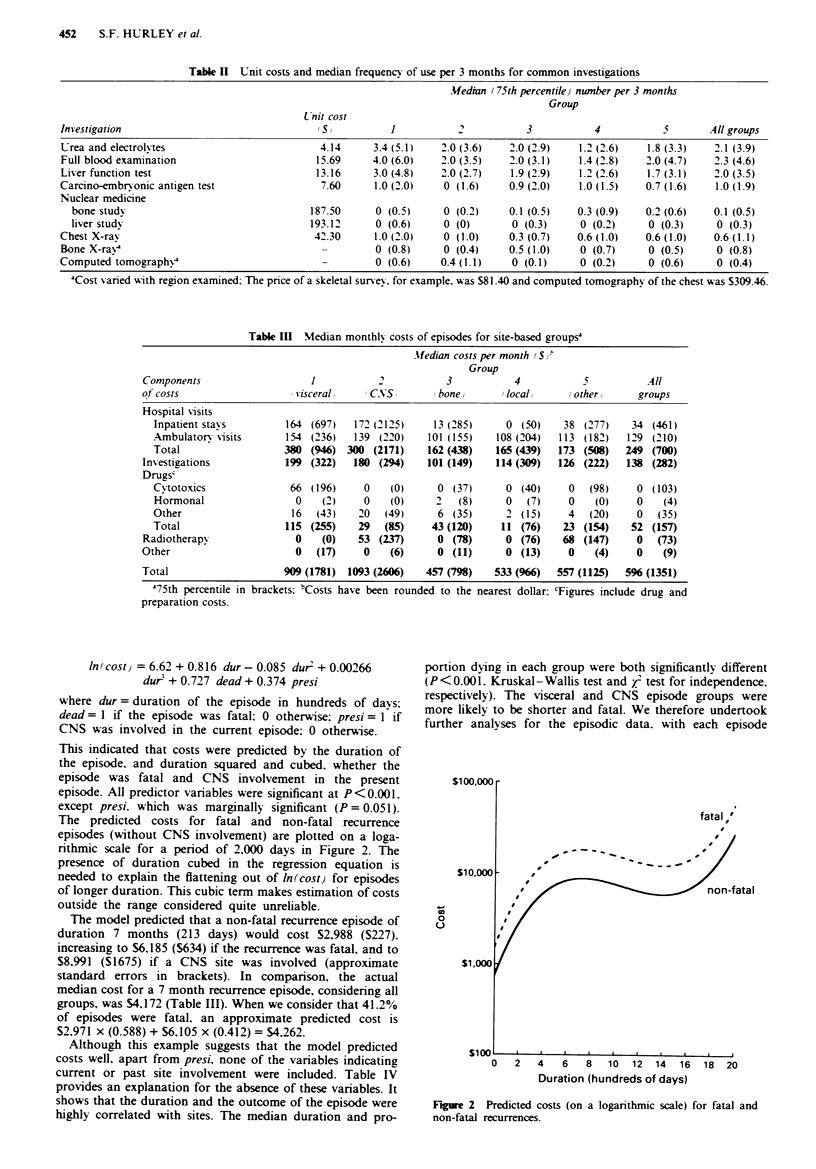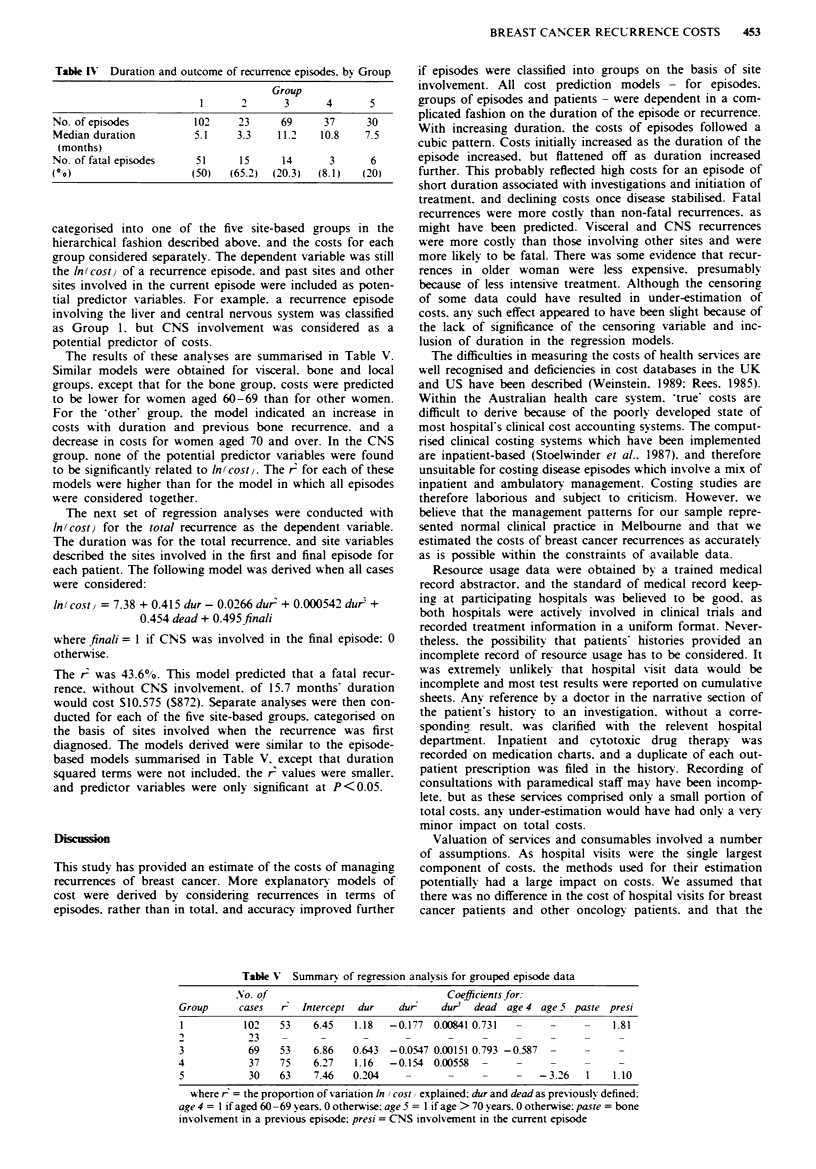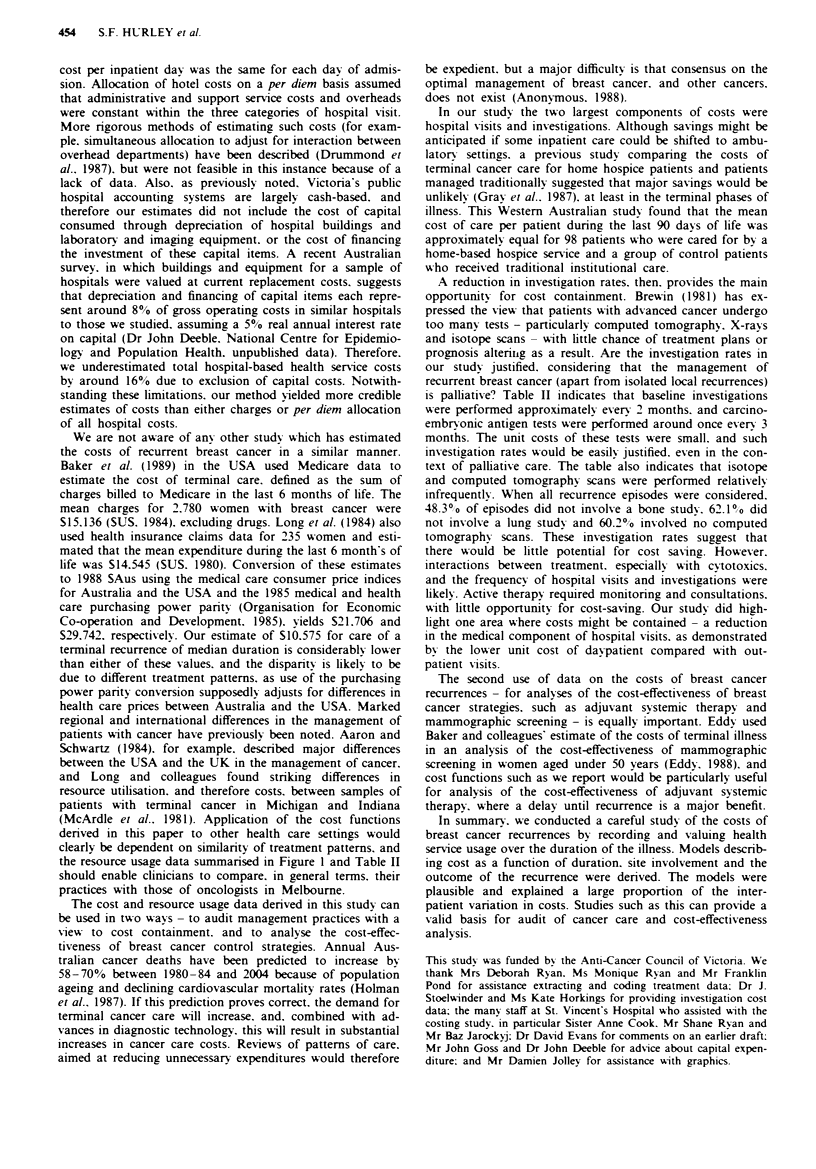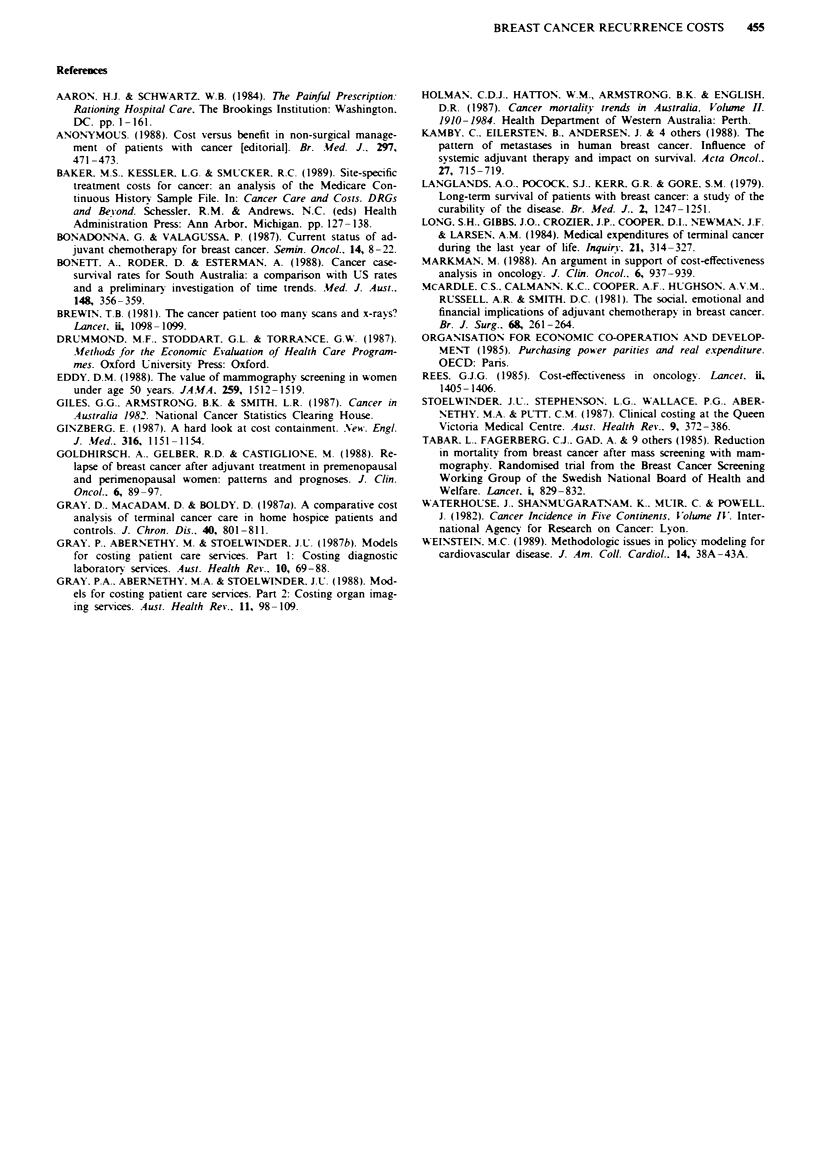# The cost of breast cancer recurrences.

**DOI:** 10.1038/bjc.1992.91

**Published:** 1992-03

**Authors:** S. F. Hurley, R. M. Huggins, R. D. Snyder, J. F. Bishop

**Affiliations:** Cancer Epidemiology Centre, Anti-Cancer Council of Victoria, Carlton South, Australia.

## Abstract

Information about the costs of recurrent breast cancer is potentially important for targeting cost containment strategies and analysing the cost-effectiveness of breast cancer control programmes. We estimated these costs by abstracting health service and consumable usage data from the medical histories of 128 patients, and valuing each of the resources used. Resource usage and costs were summarised by regarding the recurrence as a series of episodes which were categorised into five anatomical site-based groups according to the following hierarchy: visceral, central nervous system (CNS), bone, local and other. Hospital visits and investigations comprised 78% of total costs for all episodes combined, and there were significant differences between the site-based groups in the frequency of hospital visits and most investigations. Total costs were most accurately described by separate linear regression models for each group, with the natural logarithm of the cost of the episode as the dependent variable, and predictor variables including the duration of the episode, duration squared, duration cubed and a variable indicating whether the episode was fatal. Visceral and CNS episodes were associated with higher costs than the other groups and were more likely to be shorter and fatal. A fatal recurrence of duration 15.7 months (the median for our sample) was predicted to cost $10,575 (Aus + 1988; or 4,877 pounds). Reduction of the substantial costs of recurrent breast cancer is likely to be a sizable economic benefit of adjuvant systemic therapy and mammographic screening. We did not identify any major opportunities for cost containment during the management of recurrences.


*Br. J. Cancer* (1992), **65**, 449–455 © Macmillan Press Ltd., 1992

### The cost of breast cancer recurrences

S.F. Hurley[1,*], R.M. Huggins[2], R.D. Snyder[3] & J.F. Bishop[4]

[1]*Cancer Epidemiology Centre, Anti-Cancer Council of Victoria, Carlton South, Victoria 3053;* [2]*Department of Statistics, Latrobe University, Bundoora, Victoria 3083;* [3]*Oncology Unit, St Vincent's Hospital, Fitzroy, Victoria 3065;* [4]*Department of Haematology and Medical Oncology, Peter MacCallum Cancer Institute, Melbourne, Victoria 3000, Australia*

**Summary** Information about the costs of recurrent breast cancer is potentially important for targeting cost containment strategies and analysing the cost-effectiveness of breast cancer control programmes. We estimated these costs by abstracting health service and consumable usage data from the medical histories of 128 patients, and valuing each of the resources used. Resource usage and costs were summarised by regarding the recurrence as a series of episodes which were categorised into five anatomical site-based groups according to the following hierarchy: visceral, central nervous system (CNS), bone, local and other. Hospital visits and investigations comprised 78% of total costs for all episodes combined, and there were significant differences between the site-based groups in the frequency of hospital visits and most investigations. Total costs were most accurately described by separate linear regression models for each group, with the natural logarithm of the cost of the episode as the dependent variable, and predictor variables including the duration of the episode, duration squared, duration cubed and a variable indicating whether the episode was fatal. Visceral and CNS episodes were associated with higher costs than the other groups and were more likely to be shorter and fatal. A fatal recurrence of duration 15.7 months (the median for our sample) was predicted to cost $10,575 (Aus $ 1988; or £4,877). Reduction of the substantial costs of recurrent breast cancer is likely to be a sizeable economic benefit of adjuvant systemic therapy and mammographic screening. We did not identify any major opportunities for cost containment during the management of recurrences.

As health care expenditure escalates, the cost of many health services is coming under increasing scrutiny (Ginzberg, 1987) and the need to weigh the costs and benefits of cancer management, specifically, has been highlighted (Anonymous, 1988; Markman, 1988). A prerequisite for such cost-effectiveness analyses is data on health service costs during various phases of disease, but such information is often difficult to obtain. The costs of breast cancer recurrences are of particular interest because breast cancer is the most common malignancy in women in almost all countries for which incidence figures are available (Waterhouse *et al.*, 1982), and relapses occur frequently. In Australia, for example, more than 5,000 women are diagnosed with breast cancer each year (Giles *et al.*, 1987), and 5 year mortality rates of 26% have been reported (Bonett *et al.*, 1988). The fact that women with breast cancer have a higher mortality rate for 20 years or more after diagnosis has also been documented (Langlands *et al.*, 1979). The management of breast cancer recurrence is therefore a common clinical problem.

The costs of recurrent breast cancer are also relevant to studies of the cost-effectiveness of methods of controlling early breast cancer. A major advantage of adjuvant systemic therapy, for example, is delayed recurrence (Bonadonna & Valagussa, 1987). Data on the costs of treating recurrent breast cancer are therefore required to evaluate the cost-effectiveness of adjuvant systemic therapy. Similarly, such data would be required to analyse the cost-effectiveness of mammographic screening, which has been found in some studies to decrease breast cancer deaths (Tabar *et al.*, 1985), and hence would presumably decrease or delay recurrences. The aim of this study was therefore to estimate the health service costs of treating women with recurrences of breast cancer, and to investigate factors predicting costs.

*Current address: Department of Social and Preventive Medicine, Monash University, Alfred Hospital, Commercial Road, Prahran, Victoria 3181

Correspondence: Dr Susan Hurley, Senior Research Fellow, Department of Social and Preventive Medicine, Monash University, Alfred Hospital, Commercial Road, Prahran, Victoria, Australia 3181.

Received 23 April 1991; and in revised form 11 November 1991.

### Methods

The study was conducted in two major Melbourne cancer treatment hospitals, which together accounted for 58% of public hospital admissions for breast cancer in the state of Victoria in the financial year 1986-87 (Health Department Victoria, unpublished data). Public hospital services in Victoria are mostly provided free of charge to patients and are funded through a complicated state and national government cost-sharing agreement. This study was confined to hospital-based health service costs, as patients who attend public hospitals for cancer treatment in Melbourne usually receive most of their care from this source. In addition to inpatient care, hospitals provide medical consultations, investigations, drugs and paramedical services to outpatients. Patients who are terminally ill, but are not admitted to hospital, often receive nursing care from a home visiting service, but this care was not costed.

### *Identification and valuation of health service resources*

Costs were estimated by first identifying the number and type of health services used by a series of patients, valuing each type of service and then summing the costs. A series of 128 women were identified who had presented with recurrent breast cancer to the study hospitals. Sixty-three women from one hospital represented a consecutive series of patients who had received adjuvant chemotherapy in 1982–85 and had subsequently relapsed, and the 65 women from the other hospital were a consecutive series of patients who presented with a first recurrence in 1983–84. We believe our sample to be representative, as it comprised two consecutive series from hospitals with large cancer patient loads.

Information on the number and type of all health service resources used was abstracted from each patient's medical record by a trained abstractor from the time recurrence was first diagnosed until either the patient died (89 cases) or no further information was available. Six of the 89 patients who died were admitted to a hospice or a private hospital affiliated with the initial treating hospital in the weeks or months before their deaths, and in these cases the patient's medical record was abstracted at the second institution. If the patient was still alive at the time of history abstraction the data were regarded as censored, i.e. complete information about the recurrence from diagnosis to death was not available because sufficient time had not elapsed.

It was necessary to make some simplifying assumptions in order to summarise the resource usage and cost data. The concept of a recurrence episode, defined as a time period during which metastatic disease was known to involve a specific set of anatomical sites, was used. Anatomical sites were grouped into the five classifications shown in Table I because similar classifications of recurrences have been reported previously (Goldhirsch *et al.*, 1988; Kamby *et al.*, 1988) and because of an *a priori* expectation that treatment for sites within the groups would be similar. In general, treatments of choice for each site group were thought to be: hormonal and/or cytotoxic chemotherapy for visceral; radiotherapy for CNS; hormonal therapy, then cytotoxics in non-responders, for bone; surgery and radiotherapy (sometimes with cytotoxics) for local; and cytotoxics ± radiotherapy for the other group. Each episode finished when involvement of a site from another group was diagnosed, or the patient died, or history abstraction ceased. For example, an episode involving the mastectomy scar and regional nodes was classified as 'LM'. If liver metastates were diagnosed the patient was then regarded as having a new episode, classified as 'VLM'. For each episode, the number and type of each health service or consumable used, the sites involved, and the duration and reason for cessation of the episode, were noted.

A further simplification of recurrence classification was used in many analyses. Each episode was categorised in a hierarchical manner to one of five groups according to the order in which the site groupings are listed in Table I. Group 1 consisted of all episodes involving visceral sites; Group 2 consisted of all episodes that involved CNS ± other sites except visceral; Group 3 consisted of all episodes involving bone ± other sites except visceral or CNS; Group 4 consisted of all episodes involving the sites classified in Table I as local ± other sites except visceral, CNS and bone; and the last group (5) consisted of episodes involving only the 'other' sites listed in Table I. In the above example, the first episode would have been classified as Group 4, and the second episode as Group 1.

Although services and consumables were used over a period of years, starting in 1982, all resources were valued at 1988 prices (in $ Aus). Costs were not discounted because of the short time period over which they were incurred. The costs of three types of hospital visit – an inpatient day, a daypatient attendance and an outpatient attendance were calculated. A daypatient attendance was defined as a hospital visit where the patient was not admitted to a ward, but either chemotherapy was administered, or a procedure – abdominal or thoracic paracentesis, blood transfusion, lumbar puncture, urinary catheterisation or bone marrow biopsy – was performed. An outpatient attendance was an ambulatory visit which did not involve chemotherapy or a procedure. Costs for these visits were calculated by apportioning oncology unit and ward nursing staff costs to each type of visit on the basis of nursing dependency data for cancer patients and an activity study of the oncology unit; 'hotel costs' (administration, cleaning, food, power, maintenance and medical record services) were apportioned on a *per diem* basis equally to all hospital patients (irrespective of diagnosis) within the three categories of hospital visit. In this manner costs of $261.30, $88.90 and $75.80 were derived for an inpatient day, outpatient attendance and daypatient attendance, respectively. These figures excluded investigations, drugs, radiotherapy and paramedical or medical services not supplied by the oncology unit. The majority of daypatient visits were for chemotherapy, which involved less medical staff time than an outpatient visit, hence the cost of daypatient visits was lower. Capital depreciation of buildings is not included in Victoria's public hospitals' accounting systems, and was therefore not included in these costs.

**Table I** Anatomical site classifications

| Classification | Anatomical sites |
|---|---|
| Visceral (V) | Lungs, liver, pleura, abdomen, bone marrow |
| CNS (I) | Central nervous system (including eye) |
| Bone (E) | Skeletal bone |
| Local (L) | Chest wall, mastectomy scar, contralateral breast |
| Other (M) | Regional (axillary, internal mammary or supraclavicular nodes), other nodes, soft tissue, any other site. |

The clinical costing system of another Melbourne hospital (Gray *et al.*, 1987*b*, 1988) was used to value investigations. With this system, all recurrent operating expenditures are allocated to units of output on a monthly basis; capital depreciation of equipment is not included. Salary expenditures are apportioned to individual tests using the relative values for staff time; consumables are allocated on the basis of their actual costs; and overheads are allocated equally to all tests. Relative values for staff time had been derived for organ imaging and laboratory tests using the Program Evaluation and Review Technique (PERT) (Gray *et al.* 1987*b*) and the College of American Pathologists' workload recording system (Gray *et al.*, 1988), respectively. We used the mean cost of each investigation over the 6 month period, January to June 1988. Hospital overheads (administration, power, etc.) were excluded to avoid double counting.

Drug costs comprised the cost of the actual drug plus pharmacy preparation costs. Hospital wholesale drug prices were used for the former, and the latter were estimated by apportioning total annual pharmacy department costs from one hospital to each workload item, using estimated dispensing time as a relative value. The only medical staff costs included in the hospital visit costs were those of the oncology unit, and therefore Medicare benefits schedule fees (set by the Australian national health insurance organisation) were used for radiotherapy, and the medical component of any surgery and associated anaesthesia.

*Analysis*

Resource usage and costing data were entered on a customised INGRES database and analyses were performed using the Structured Query Language (SQL) and the statistical computing package MINITAB. Costs were disaggregated into hospital visits (outpatients and daypatients were combined and summarised as ambulatory visits), investigations, radiotherapy, drugs, and other costs. Drugs costs were subdivided into cytotoxics, hormonal drugs, and other drugs, and each group was further subdivided into the costs of the drug itself and preparation costs.

A large number of factors had the potential to affect costs, including duration of the recurrence, anatomic sites involved and the patient's age. It was not feasible to present multiple tabulations of costs categorised according to these factors, and a regression approach was therefore used to determine the factors affecting total costs. Stepwise regression analysis was conducted with both $F$ to enter and $F$ to leave set to four. The dependent variable was taken to be the natural logarithm of cost, $ln(cost)$. Two separate sets of analyses were performed, the first with $ln(cost)$ for each recurrence episode as the dependent variable, and the second with $ln(cost)$ for the total recurrence (i.e. per patient rather than per episode). The predictor variables considered included duration, duration squared and duration cubed, whether or not the recurrence episode (or recurrence for the second analysis) was fatal, five age categories (<39, 40–49, 50–59, 60–69, and >70 years), past and present sites of recurrence, and whether the episodes (or recurrence) was censored. The choice of transformation for the dependent variable, and inclusion of duration squared and cubed as potential predictor variables, was based on an observed polynomial pattern when $ln(cost)$ was plotted against the duration of the recurrence episode. Inclusion of past and present sites of recurrence as potential predictor variables allowed for the possibility that the costs of an episode would vary with past site involvement or any of the sites involved in the current episode. Duration was measured in hundreds of days for the regression analyses.

### Results

### *Patients and recurrence details*

The mean age of patients at the onset of recurrence was 52.9 years (95% CI 51.1, 54.8; median 54.4). The mean duration from the time breast cancer was first diagnosed until recurrence occurred was 27.5 months (95% CI 23.8, 31.1; median 21.7). The 128 women experienced 261 recurrence episodes, a mean of 2.07 per patient (maximum 5). Resource usage, costs and duration of the recurrence episodes all had skewed distributions, and therefore medians were mainly used as measures of central tendency. The median duration of the total recurrence per patient was 17.8 months (15.7 months for those who died, 29.5 months for those for whom data were censored). Of the 261 recurrence episodes, 102 were categorised as Group 1 (visceral), 23 were Group 2 (CNS), 69 were Group 3 (bone), 37 were Group 4 (local), and 30 were Group 5 (other).

### *Resource usage patterns*

For each episode, the number of each type of health service used per 3 months was calculated. If the duration of the episode was less than 3 months, the actual number of services was used for the analysis. The usage patterns for each type of service were compared for the different site-based groups using the Kruskal-Wallis non-parametric rank test. The number of inpatient admissions, inpatient bed days (the sum of the lengths of stay for all admissions) and number of daypatient visits differed significantly between the five groups ($P = 0.0031$, 0.0011 and 0.0002, respectively), but the number of outpatients visits did not. The distributions of inpatient days, outpatients visits and daypatient attendances are shown as box plots in Figure 1.

The unit costs, and median and 75th percentiles of the number of tests per 3 months for the most frequently used investigations are summarised in Table II. There were significant differences between groups in the median frequency of use for all tests, except carcino-embryonic antigen assays and nuclear medicine investigations.

There were also differences between the site groups in radiotherapy and cytotoxic drug treatment patterns over the duration of the episode. Radiotherapy was used more commonly in Group 5 episodes (which included sites with nodal involvement), and least frequently during Group 1 episodes. One or more radiotherapy treatments were used during 21.6%, 52.2%, 49.3%, 43.2% and 70% of episodes from Groups 1–5 respectively. Cytotoxics were more commonly prescribed for visceral episodes, followed by 'other', local, bone and CNS. Approximately 65% of visceral episodes involved treatment with a cytotoxic drug, compared with 46.7% of episodes from the 'other' group, 42% of bone, 27% of local, and 17.4% of CNS episodes. Tamoxifen was prescribed during 52.2% of bone, 28% of visceral, 26.7% of 'other', 21.6% of local and 8.7% of CNS episodes. These treatment patterns were therefore similar to the expected treatment patterns described in the Methods section.

### *Costs*

The median (75th percentile) cost per episode for all episode groups combined was $4.295 (S10.124) and the median cost per recurrence was S11.349 (S18.258). For the 89 patients who died, the median cost per recurrence was S11.948 (S18.771). The median and 75th percentiles for the total and component costs per episode per month, for each of the five groups are summarised in Table III. The total monthly costs

**Figure 1** Box plots of the number of inpatient days, outpatient attendances and daypatient attendances per 3 months for each of the episode groups. These plots display data values from the 25th to the 75th percentile as a box, with the median value as a horizontal bar. The range of the remaining data is represented by bars which extend from the box to the nearest data point below a distance 1.5 times the interquartile range. Observations lying outside this range are shown as individual points.

for visceral and CNS recurrence episodes were substantially higher than for bone, local or 'other' recurrences. As noted above, the cost distributions were skewed to the right, due to a few high cost outliers, and therefore mean costs were higher than median costs. For Groups 1–5, and all episode groups combined, mean total monthly costs were S2.148, S2.163, S723, S836, S838 and S1.436, respectively.

For each group, hospital visits and investigations were the largest and second largest components of total costs, respectively. Hospital visits comprised 53.8% of mean total costs, and investigations 24.2% for all episodes combined. Preparation costs comprised only 15.7% of mean monthly total drug costs. Radiotherapy costs were a very small component of total costs for all groups except Group 2 (CNS) and Group 5.

### *Regression analyses*

The first regression analysis, where recurrences were considered in terms of episodes, and all episodes were included in the analysis, yielded the following equation (with $r^2 = 48.9\%$).

**Table II** Unit costs and median frequency of use per 3 months for common investigations

| Investigation | Unit cost (\$) | Median (75th percentile) number per 3 months Group 1 | 2 | 3 | 4 | 5 | All groups |
|---|---|---|---|---|---|---|---|
| Urea and electrolytes | 4.14 | 3.4 (5.1) | 2.0 (3.6) | 2.0 (2.9) | 1.2 (2.6) | 1.8 (3.3) | 2.1 (3.9) |
| Full blood examination | 15.69 | 4.0 (6.0) | 2.0 (3.5) | 2.0 (3.1) | 1.4 (2.8) | 2.0 (4.7) | 2.3 (4.6) |
| Liver function test | 13.16 | 3.0 (4.8) | 2.0 (2.7) | 1.9 (2.9) | 1.2 (2.6) | 1.7 (3.1) | 2.0 (3.5) |
| Carcino-embryonic antigen test | 7.60 | 1.0 (2.0) | 0 (1.6) | 0.9 (2.0) | 1.0 (1.5) | 0.7 (1.6) | 1.0 (1.9) |
| Nuclear medicine | | | | | | | |
| bone study | 187.50 | 0 (0.5) | 0 (0.2) | 0.1 (0.5) | 0.3 (0.9) | 0.2 (0.6) | 0.1 (0.5) |
| liver study | 193.12 | 0 (0.6) | 0 (0) | 0 (0.3) | 0 (0.2) | 0 (0.3) | 0 (0.3) |
| Chest X-ray | 42.30 | 1.0 (2.0) | 0 (1.0) | 0.3 (0.7) | 0.6 (1.0) | 0.6 (1.0) | 0.6 (1.1) |
| Bone X-ray[a] | – | 0 (0.8) | 0 (0.4) | 0.5 (1.0) | 0 (0.7) | 0 (0.5) | 0 (0.8) |
| Computed tomography[a] | – | 0 (0.6) | 0.4 (1.1) | 0 (0.1) | 0 (0.2) | 0 (0.6) | 0 (0.4) |

[a]Cost varied with region examined: The price of a skeletal survey, for example, was \$81.40 and computed tomography of the chest was \$309.46.

**Table III** Median monthly costs of episodes for site-based groups[a]

| Components of costs | Median costs per month (\$)[b] Group 1 (visceral) | 2 (CNS) | 3 (bone) | 4 (local) | 5 (other) | All groups |
|---|---|---|---|---|---|---|
| Hospital visits | | | | | | |
| Inpatient stays | 164 (697) | 172 (2125) | 13 (285) | 0 (50) | 38 (277) | 34 (461) |
| Ambulatory visits | 154 (236) | 139 (220) | 101 (155) | 108 (204) | 113 (182) | 129 (210) |
| Total | **380 (946)** | **300 (2171)** | **162 (438)** | **165 (439)** | **173 (508)** | **249 (700)** |
| Investigations | **199 (322)** | **180 (294)** | **101 (149)** | **114 (309)** | **126 (222)** | **138 (282)** |
| Drugs[c] | | | | | | |
| Cytotoxics | 66 (196) | 0 (0) | 0 (37) | 0 (40) | 0 (98) | 0 (103) |
| Hormonal | 0 (2) | 0 (0) | 2 (8) | 0 (7) | 0 (0) | 0 (4) |
| Other | 16 (43) | 20 (49) | 6 (35) | 2 (15) | 4 (20) | 0 (35) |
| Total | **115 (255)** | **29 (85)** | **43 (120)** | **11 (76)** | **23 (154)** | **52 (157)** |
| Radiotherapy | **0 (0)** | **53 (237)** | **0 (78)** | **0 (76)** | **68 (147)** | **0 (73)** |
| Other | **0 (17)** | **0 (6)** | **0 (11)** | **0 (13)** | **0 (4)** | **0 (9)** |
| Total | **909 (1781)** | **1093 (2606)** | **457 (798)** | **533 (966)** | **557 (1125)** | **596 (1351)** |

[a]75th percentile in brackets; [b]Costs have been rounded to the nearest dollar; [c]Figures include drug and preparation costs.

$$ln(cost) = 6.62 + 0.816\ dur - 0.085\ dur^2 + 0.00266\ dur^3 + 0.727\ dead + 0.374\ presi$$

where *dur* = duration of the episode in hundreds of days; *dead* = 1 if the episode was fatal; 0 otherwise; *presi* = 1 if CNS was involved in the current episode; 0 otherwise.

This indicated that costs were predicted by the duration of the episode, and duration squared and cubed, whether the episode was fatal and CNS involvement in the present episode. All predictor variables were significant at $P<0.001$, except *presi*, which was marginally significant ($P = 0.051$). The predicted costs for fatal and non-fatal recurrence episodes (without CNS involvement) are plotted on a logarithmic scale for a period of 2,000 days in Figure 2. The presence of duration cubed in the regression equation is needed to explain the flattening out of *ln(cost)* for episodes of longer duration. This cubic term makes estimation of costs outside the range considered quite unreliable.

The model predicted that a non-fatal recurrence episode of duration 7 months (213 days) would cost \$2,988 (\$227), increasing to \$6,185 (\$634) if the recurrence was fatal, and to \$8,991 (\$1675) if a CNS site was involved (approximate standard errors in brackets). In comparison, the actual median cost for a 7 month recurrence episode, considering all groups, was \$4,172 (Table III). When we consider that 41.2% of episodes were fatal, an approximate predicted cost is \$2,971 × (0.588) + \$6,105 × (0.412) = \$4,262.

Although this example suggests that the model predicted costs well, apart from *presi*, none of the variables indicating current or past site involvement were included. Table IV provides an explanation for the absence of these variables. It shows that the duration and the outcome of the episode were highly correlated with sites. The median duration and proportion dying in each group were both significantly different ($P<0.001$, Kruskal–Wallis test and $\chi^2$ test for independence, respectively). The visceral and CNS episode groups were more likely to be shorter and fatal. We therefore undertook further analyses for the episodic data, with each episode

**Figure 2** Predicted costs (on a logarithmic scale) for fatal and non-fatal recurrences.

**Table IV** Duration and outcome of recurrence episodes, by Group

| | Group | | | | |
|---|---|---|---|---|---|
| | 1 | 2 | 3 | 4 | 5 |
| No. of episodes | 102 | 23 | 69 | 37 | 30 |
| Median duration (months) | 5.1 | 3.3 | 11.2 | 10.8 | 7.5 |
| No. of fatal episodes | 51 | 15 | 14 | 3 | 6 |
| (%) | (50) | (65.2) | (20.3) | (8.1) | (20) |

categorised into one of the five site-based groups in the hierarchical fashion described above, and the costs for each group considered separately. The dependent variable was still the *ln(cost)* of a recurrence episode, and past sites and other sites involved in the current episode were included as potential predictor variables. For example, a recurrence episode involving the liver and central nervous system was classified as Group 1, but CNS involvement was considered as a potential predictor of costs.

The results of these analyses are summarised in Table V. Similar models were obtained for visceral, bone and local groups, except that for the bone group, costs were predicted to be lower for women aged 60–69 than for other women. For the 'other' group, the model indicated an increase in costs with duration and previous bone recurrence, and a decrease in costs for women aged 70 and over. In the CNS group, none of the potential predictor variables were found to be significantly related to *ln(cost)*. The $r^2$ for each of these models were higher than for the model in which all episodes were considered together.

The next set of regression analyses were conducted with *ln(cost)* for the *total* recurrence as the dependent variable. The duration was for the total recurrence, and site variables described the sites involved in the first and final episode for each patient. The following model was derived when all cases were considered:

$$ln(cost) = 7.38 + 0.415\,dur - 0.0266\,dur^2 + 0.000542\,dur^3 + 0.454\,dead + 0.495\,finali$$

where *finali* = 1 if CNS was involved in the final episode; 0 otherwise.

The $r^2$ was 43.6%. This model predicted that a fatal recurrence, without CNS involvement, of 15.7 months' duration would cost $10,575 ($872). Separate analyses were then conducted for each of the five site-based groups, categorised on the basis of sites involved when the recurrence was first diagnosed. The models derived were similar to the episode-based models summarised in Table V, except that duration squared terms were not included, the $r^2$ values were smaller, and predictor variables were only significant at $P<0.05$.

### Discussion

This study has provided an estimate of the costs of managing recurrences of breast cancer. More explanatory models of cost were derived by considering recurrences in terms of episodes, rather than in total, and accuracy improved further if episodes were classified into groups on the basis of site involvement. All cost prediction models – for episodes, groups of episodes and patients – were dependent in a complicated fashion on the duration of the episode or recurrence. With increasing duration, the costs of episodes followed a cubic pattern. Costs initially increased as the duration of the episode increased, but flattened off as duration increased further. This probably reflected high costs for an episode of short duration associated with investigations and initiation of treatment, and declining costs once disease stabilised. Fatal recurrences were more costly than non-fatal recurrences, as might have been predicted. Visceral and CNS recurrences were more costly than those involving other sites and were more likely to be fatal. There was some evidence that recurrences in older woman were less expensive, presumably because of less intensive treatment. Although the censoring of some data could have resulted in under-estimation of costs, any such effect appeared to have been slight because of the lack of significance of the censoring variable and inclusion of duration in the regression models.

The difficulties in measuring the costs of health services are well recognised and deficiencies in cost databases in the UK and US have been described (Weinstein, 1989; Rees, 1985). Within the Australian health care system, 'true' costs are difficult to derive because of the poorly developed state of most hospital's clinical cost accounting systems. The computrised clinical costing systems which have been implemented are inpatient-based (Stoelwinder *et al.*, 1987), and therefore unsuitable for costing disease episodes which involve a mix of inpatient and ambulatory management. Costing studies are therefore laborious and subject to criticism. However, we believe that the management patterns for our sample represented normal clinical practice in Melbourne and that we estimated the costs of breast cancer recurrences as accurately as is possible within the constraints of available data.

Resource usage data were obtained by a trained medical record abstractor, and the standard of medical record keeping at participating hospitals was believed to be good, as both hospitals were actively involved in clinical trials and recorded treatment information in a uniform format. Nevertheless, the possibility that patients' histories provided an incomplete record of resource usage has to be considered. It was extremely unlikely that hospital visit data would be incomplete and most test results were reported on cumulative sheets. Any reference by a doctor in the narrative section of the patient's history to an investigation, without a corresponding result, was clarified with the relevent hospital department. Inpatient and cytotoxic drug therapy was recorded on medication charts, and a duplicate of each outpatient prescription was filed in the history. Recording of consultations with paramedical staff may have been incomplete, but as these services comprised only a small portion of total costs, any under-estimation would have had only a very minor impact on total costs.

Valuation of services and consumables involved a number of assumptions. As hospital visits were the single largest component of costs, the methods used for their estimation potentially had a large impact on costs. We assumed that there was no difference in the cost of hospital visits for breast cancer patients and other oncology patients, and that the

**Table V** Summary of regression analysis for grouped episode data

| Group | No. of cases | $r^2$ | Intercept | dur | $dur^2$ | $dur^3$ | dead | age 4 | age 5 | paste | presi |
|---|---|---|---|---|---|---|---|---|---|---|---|
| | | | | | Coefficients for: | | | | | | |
| 1 | 102 | 53 | 6.45 | 1.18 | −0.177 | 0.00841 | 0.731 | – | – | – | 1.81 |
| 2 | 23 | – | – | – | – | – | – | – | – | – | – |
| 3 | 69 | 53 | 6.86 | 0.643 | −0.0547 | 0.00151 | 0.793 | −0.587 | – | – | – |
| 4 | 37 | 75 | 6.27 | 1.16 | −0.154 | 0.00558 | – | – | – | – | – |
| 5 | 30 | 63 | 7.46 | 0.204 | – | – | – | – | −3.26 | 1 | 1.10 |

where $r^2$ = the proportion of variation *ln (cost)* explained; *dur* and *dead* as previously defined; *age 4* = 1 if aged 60–69 years, 0 otherwise; *age 5* = 1 if age > 70 years, 0 otherwise; *paste* = bone involvement in a previous episode; *presi* = CNS involvement in the current episode cost per inpatient day was the same for each day of admission. Allocation of hotel costs on a *per diem* basis assumed that administrative and support service costs and overheads were constant within the three categories of hospital visit. More rigorous methods of estimating such costs (for example, simultaneous allocation to adjust for interaction between overhead departments) have been described (Drummond *et al.*, 1987), but were not feasible in this instance because of a lack of data. Also, as previously noted, Victoria's public hospital accounting systems are largely cash-based, and therefore our estimates did not include the cost of capital consumed through depreciation of hospital buildings and laboratory and imaging equipment, or the cost of financing the investment of these capital items. A recent Australian survey, in which buildings and equipment for a sample of hospitals were valued at current replacement costs, suggests that depreciation and financing of capital items each represent around 8% of gross operating costs in similar hospitals to those we studied, assuming a 5% real annual interest rate on capital (Dr John Deeble, National Centre for Epidemiology and Population Health, unpublished data). Therefore, we underestimated total hospital-based health service costs by around 16% due to exclusion of capital costs. Notwithstanding these limitations, our method yielded more credible estimates of costs than either charges or *per diem* allocation of all hospital costs.

We are not aware of any other study which has estimated the costs of recurrent breast cancer in a similar manner. Baker *et al.* (1989) in the USA used Medicare data to estimate the cost of terminal care, defined as the sum of charges billed to Medicare in the last 6 months of life. The mean charges for 2,780 women with breast cancer were $15,136 ($US, 1984), excluding drugs. Long *et al.* (1984) also used health insurance claims data for 235 women and estimated that the mean expenditure during the last 6 month's of life was $14,545 ($US, 1980). Conversion of these estimates to 1988 $Aus using the medical care consumer price indices for Australia and the USA and the 1985 medical and health care purchasing power parity (Organisation for Economic Co-operation and Development, 1985), yields $21,706 and $29,742, respectively. Our estimate of $10,575 for care of a terminal recurrence of median duration is considerably lower than either of these values, and the disparity is likely to be due to different treatment patterns, as use of the purchasing power parity conversion supposedly adjusts for differences in health care prices between Australia and the USA. Marked regional and international differences in the management of patients with cancer have previously been noted. Aaron and Schwartz (1984), for example, described major differences between the USA and the UK in the management of cancer, and Long and colleagues found striking differences in resource utilisation, and therefore costs, between samples of patients with terminal cancer in Michigan and Indiana (McArdle *et al.*, 1981). Application of the cost functions derived in this paper to other health care settings would clearly be dependent on similarity of treatment patterns, and the resource usage data summarised in Figure 1 and Table II should enable clinicians to compare, in general terms, their practices with those of oncologists in Melbourne.

The cost and resource usage data derived in this study can be used in two ways – to audit management practices with a view to cost containment, and to analyse the cost-effectiveness of breast cancer control strategies. Annual Australian cancer deaths have been predicted to increase by 58–70% between 1980–84 and 2004 because of population ageing and declining cardiovascular mortality rates (Holman *et al.*, 1987). If this prediction proves correct, the demand for terminal cancer care will increase, and, combined with advances in diagnostic technology, this will result in substantial increases in cancer care costs. Reviews of patterns of care, aimed at reducing unnecessary expenditures would therefore be expedient, but a major difficulty is that consensus on the optimal management of breast cancer, and other cancers, does not exist (Anonymous, 1988).

In our study the two largest components of costs were hospital visits and investigations. Although savings might be anticipated if some inpatient care could be shifted to ambulatory settings, a previous study comparing the costs of terminal cancer care for home hospice patients and patients managed traditionally suggested that major savings would be unlikely (Gray *et al.*, 1987), at least in the terminal phases of illness. This Western Australian study found that the mean cost of care per patient during the last 90 days of life was approximately equal for 98 patients who were cared for by a home-based hospice service and a group of control patients who received traditional institutional care.

A reduction in investigation rates, then, provides the main opportunity for cost containment. Brewin (1981) has expressed the view that patients with advanced cancer undergo too many tests – particularly computed tomography, X-rays and isotope scans – with little chance of treatment plans or prognosis altering as a result. Are the investigation rates in our study justified, considering that the management of recurrent breast cancer (apart from isolated local recurrences) is palliative? Table II indicates that baseline investigations were performed approximately every 2 months, and carcinoembryonic antigen tests were performed around once every 3 months. The unit costs of these tests were small, and such investigation rates would be easily justified, even in the context of palliative care. The table also indicates that isotope and computed tomography scans were performed relatively infrequently. When all recurrence episodes were considered, 48.3% of episodes did not involve a bone study, 62.1% did not involve a lung study and 60.2% involved no computed tomography scans. These investigation rates suggest that there would be little potential for cost saving. However, interactions between treatment, especially with cytotoxics, and the frequency of hospital visits and investigations were likely. Active therapy required monitoring and consultations, with little opportunity for cost-saving. Our study did highlight one area where costs might be contained – a reduction in the medical component of hospital visits, as demonstrated by the lower unit cost of daypatient compared with outpatient visits.

The second use of data on the costs of breast cancer recurrences – for analyses of the cost-effectiveness of breast cancer strategies, such as adjuvant systemic therapy and mammographic screening – is equally important. Eddy used Baker and colleagues' estimate of the costs of terminal illness in an analysis of the cost-effectiveness of mammographic screening in women aged under 50 years (Eddy, 1988), and cost functions such as we report would be particularly useful for analysis of the cost-effectiveness of adjuvant systemic therapy, where a delay until recurrence is a major benefit.

In summary, we conducted a careful study of the costs of breast cancer recurrences by recording and valuing health service usage over the duration of the illness. Models describing cost as a function of duration, site involvement and the outcome of the recurrence were derived. The models were plausible and explained a large proportion of the interpatient variation in costs. Studies such as this can provide a valid basis for audit of cancer care and cost-effectiveness analysis.

This study was funded by the Anti-Cancer Council of Victoria. We thank Mrs Deborah Ryan, Ms Monique Ryan and Mr Franklin Pond for assistance extracting and coding treatment data; Dr J. Stoelwinder and Ms Kate Horkings for providing investigation cost data; the many staff at St. Vincent's Hospital who assisted with the costing study, in particular Sister Anne Cook, Mr Shane Ryan and Mr Baz Jarockyj; Dr David Evans for comments on an earlier draft; Mr John Goss and Dr John Deeble for advice about capital expenditure; and Mr Damien Jolley for assistance with graphics.

**References**

AARON, H.J. & SCHWARTZ, W.B. (1984). *The Painful Prescription: Rationing Hospital Care*. The Brookings Institution: Washington, DC. pp. 1-161.

ANONYMOUS. (1988). Cost versus benefit in non-surgical management of patients with cancer [editorial]. *Br. Med. J.*, **297**, 471-473.

BAKER, M.S., KESSLER, L.G. & SMUCKER, R.C. (1989). Site-specific treatment costs for cancer: an analysis of the Medicare Continuous History Sample File. In: *Cancer Care and Costs. DRGs and Beyond*. Schessler, R.M. & Andrews, N.C. (eds) Health Administration Press: Ann Arbor, Michigan. pp. 127-138.

BONADONNA, G. & VALAGUSSA, P. (1987). Current status of adjuvant chemotherapy for breast cancer. *Semin. Oncol.*, **14**, 8-22.

BONETT, A., RODER, D. & ESTERMAN, A. (1988). Cancer case-survival rates for South Australia: a comparison with US rates and a preliminary investigation of time trends. *Med. J. Aust.*, **148**, 356-359.

BREWIN, T.B. (1981). The cancer patient too many scans and x-rays? *Lancet*, **ii**, 1098-1099.

DRUMMOND, M.F., STODDART, G.L. & TORRANCE, G.W. (1987). *Methods for the Economic Evaluation of Health Care Programmes*. Oxford University Press: Oxford.

EDDY, D.M. (1988). The value of mammography screening in women under age 50 years. *JAMA*, **259**, 1512-1519.

GILES, G.G., ARMSTRONG, B.K. & SMITH, L.R. (1987). *Cancer in Australia 1982*. National Cancer Statistics Clearing House.

GINZBERG, E. (1987). A hard look at cost containment. *New Engl. J. Med.*, **316**, 1151-1154.

GOLDHIRSCH, A., GELBER, R.D. & CASTIGLIONE, M. (1988). Relapse of breast cancer after adjuvant treatment in premenopausal and perimenopausal women: patterns and prognoses. *J. Clin. Oncol.*, **6**, 89-97.

GRAY, D., MACADAM, D. & BOLDY, D. (1987*a*). A comparative cost analysis of terminal cancer care in home hospice patients and controls. *J. Chron. Dis.*, **40**, 801-811.

GRAY, P., ABERNETHY, M. & STOELWINDER, J.U. (1987*b*). Models for costing patient care services. Part 1: Costing diagnostic laboratory services. *Aust. Health Rev.*, **10**, 69-88.

GRAY, P.A., ABERNETHY, M.A. & STOELWINDER, J.U. (1988). Models for costing patient care services. Part 2: Costing organ imaging services. *Aust. Health Rev.*, **11**, 98-109.

HOLMAN, C.D.J., HATTON, W.M., ARMSTRONG, B.K. & ENGLISH, D.R. (1987). *Cancer mortality trends in Australia, Volume II, 1910-1984*. Health Department of Western Australia: Perth.

KAMBY, C., EILERSTEN, B., ANDERSEN, J. & 4 others (1988). The pattern of metastases in human breast cancer. Influence of systemic adjuvant therapy and impact on survival. *Acta Oncol.*, **27**, 715-719.

LANGLANDS, A.O., POCOCK, S.J., KERR, G.R. & GORE, S.M. (1979). Long-term survival of patients with breast cancer: a study of the curability of the disease. *Br. Med. J.*, **2**, 1247-1251.

LONG, S.H., GIBBS, J.O., CROZIER, J.P., COOPER, D.I., NEWMAN, J.F. & LARSEN, A.M. (1984). Medical expenditures of terminal cancer during the last year of life. *Inquiry*, **21**, 314-327.

MARKMAN, M. (1988). An argument in support of cost-effectiveness analysis in oncology. *J. Clin. Oncol.*, **6**, 937-939.

MCARDLE, C.S., CALMANN, K.C., COOPER, A.F., HUGHSON, A.V.M., RUSSELL, A.R. & SMITH, D.C. (1981). The social, emotional and financial implications of adjuvant chemotherapy in breast cancer. *Br. J. Surg.*, **68**, 261-264.

ORGANISATION FOR ECONOMIC CO-OPERATION AND DEVELOPMENT (1985). *Purchasing power parities and real expenditure*. OECD: Paris.

REES, G.J.G. (1985). Cost-effectiveness in oncology. *Lancet*, **ii**, 1405-1406.

STOELWINDER, J.U., STEPHENSON, L.G., WALLACE, P.G., ABERNETHY, M.A. & PUTT, C.M. (1987). Clinical costing at the Queen Victoria Medical Centre. *Aust. Health Rev.*, **9**, 372-386.

TABAR, L., FAGERBERG, C.J., GAD, A. & 9 others (1985). Reduction in mortality from breast cancer after mass screening with mammography. Randomised trial from the Breast Cancer Screening Working Group of the Swedish National Board of Health and Welfare. *Lancet*, **i**, 829-832.

WATERHOUSE, J., SHANMUGARATNAM, K., MUIR, C. & POWELL, J. (1982). *Cancer Incidence in Five Continents, Volume IV*. International Agency for Research on Cancer: Lyon.

WEINSTEIN, M.C. (1989). Methodologic issues in policy modeling for cardiovascular disease. *J. Am. Coll. Cardiol.*, **14**, 38A-43A.